# Electronic Spectroscopy of Ovalene: Reassignment of the *S*_2_(*B*_3u_)– *S*_0_(*A*_g_) Transition

**DOI:** 10.1021/acs.jpclett.4c02494

**Published:** 2024-10-16

**Authors:** Isabelle Weber, Johanna Langner, Henryk A. Witek, Yuan-Pern Lee

**Affiliations:** † Department of Applied Chemistry and Institute of Molecular Science, National Yang Ming Chiao Tung University, Hsinchu 300093, Taiwan; ‡ Center for Emergent Functional Matter Science, National Yang Ming Chiao Tung University, Hsinchu 300093, Taiwan

## Abstract

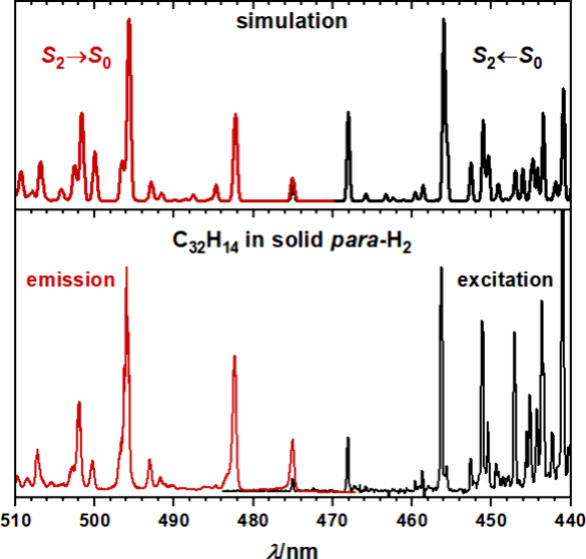

As part of our experiments to characterize solid *para*-hydrogen (*para*-H_2_) as a matrix host for electronic spectroscopy with potential applications in the ongoing quest for the carriers of the diffuse interstellar bands (DIB), we studied dispersed fluorescence and fluorescence excitation spectra of ovalene (C_32_H_14_), a planar polycyclic aromatic hydrocarbon (PAH) of *D*_2h_ symmetry. Although generally in good agreement with previously reported data for jet-cooled C_32_H_14_, our results, in conjunction with quantum-chemical calculations, indicate that the observed spectral progressions are associated with the *S*_2_(*B*_3u_)–*S*_0_(*A*_g_) electronic transition instead of the originally assigned *S*_1_–*S*_0_ transition for C_32_H_14_ in a supersonic jet, and that the previously reported origin band was misassigned and should be located at ∼21050 cm^–1^. The reassignment is further supported by the comparably long fluorescence lifetime of ∼1.7 μs. From an analysis of spectral features located >1600 cm^–1^ in the fluorescence excitation spectrum, we estimate an *S*_2_(*B*_3u_)–*S*_3_(*B*_1g_) energy gap of ∼350 cm^–1^.

The search for the carriers of the diffuse interstellar bands (DIB), absorption features observed on various astronomical sightlines, is an ongoing quest in astronomy and astrochemistry. Whilst well over 500 DIB have been identified with their maximum density in the range 500–700 nm,^1,2^ only the buckminsterfullerene cation C_60_^+^ has been confirmed as the carrier of six DIB in the near-infrared region.^3,4^ The search for the DIB carriers is hampered by a lack of reference spectra suitable for comparison to astronomical observations. Promising candidates are large polycyclic aromatic hydrocarbons (PAH) with number of carbon atoms ≥42 and their derivatives,^5−7^ but the gas-phase spectra under cold conditions are challenging to obtain due to their low vapor pressures. Matrix isolation methods provide an alternative as significantly smaller amounts of samples isolated in the solid are required; however, the interactions between the matrix host and the guest molecules may affect line positions. Previously frequently employed in IR absorption studies, the quantum solid *para*-H_2_ has shown promising properties such as small infrared matrix shifts (≤1%) and narrow line widths, indicating only weak interactions with the isolated guest molecules.^8−10^ One would expect the matrix shifts of electronic transitions to be small and consistent in solid *para-*H_2_, but experimental data are scarce. Over the past years, we recorded dispersed fluorescence and fluorescence excitation spectra of several PAH isolated in solid *para*-H_2_ to assess the properties of *para*-H_2_ as a matrix host for electronic spectroscopy.^11−13^

Ovalene, C_32_H_14_, a planar, *peri*-condensed PAH of *D*_2*h*_ symmetry shown in Figure 1, was first synthesized by Clar in 1948.^14^ Over the past decades, C_32_H_14_ and its derivatives have drawn increasing attention as a potential carrier of DIB,^15−17^ as an intermediate in soot formation,^18^ as a model for graphene and graphene nanoflakes in quantum-chemical studies,^19,20^ and, with its planarity and extended π-electron system, as a potential functional material for nanoelectronics,^21^ organic light-emitting devices (OLED),^22^ and quantum dots.^23^ Experimental studies characterizing the fundamental spectroscopic, physical, and chemical properties of C_32_H_14_, which could serve as benchmarks for calculations, however, are scarce.

**Figure 1:**
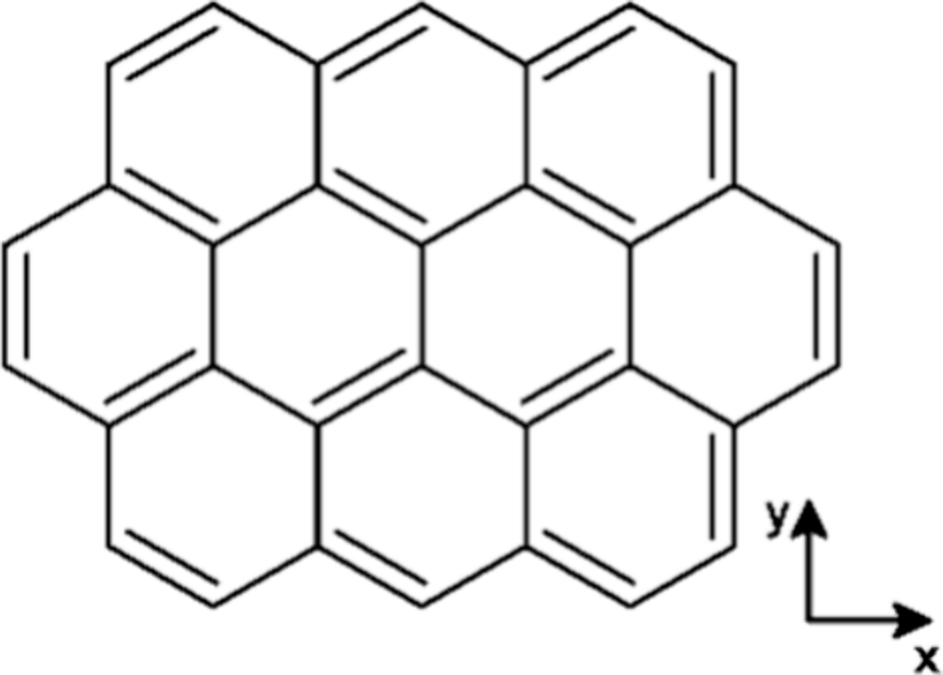
Structure of ovalene (C_32_H_14_) and orientation of Cartesian coordinates.

Early work reported broad bands of electronic absorption and dispersed fluorescence spectra of C_32_H_14_ in organic solvents over wide temperature and pressure ranges,^14,24−26^ and emphasized the influence of coupling of the observed lowest excited electronic states *S*_1_ and *S*_2_ near 465.0 and 456.0 nm, respectively, facilitated by a small *S*_2_–*S*_1_ energy gap. Anomalous fluorescence from *S*_2_ was reported by Johnson and Offen^25^ and Kropp and Stanley;^24^ the latter estimated an *S*_2_–*S*_1_ energy gap of ∼450 cm^–1^ from temperature-dependent emission measurements. To the best of our knowledge, only three groups have reported electronic spectra of C_32_H_14_ obtained by matrix-isolation or jet-expansion methods, which are not subject to severe solvent interactions.

In search of absorption bands of coronene, ovalene, and their single-charged cations in astronomical observations, Ehrenfreund et al.^15,16^ recorded absorption spectra of these compounds in solid Ne. A similar work with many other PAH covering an extended wavelength range was published several years later by Ruiterkamp et al.^27^ Individual peak positions in the region 215–455 nm were reported and related to the three band systems at ∼215.8 nm, 270–340 nm, and 340–430 nm identified by Clar,^28^ but no vibronic analysis was provided. In a series of two publications, Amirav et al.^29,30^ analyzed fluorescence lifetimes, emission, and excitation spectra of C_32_H_14_ in a supersonic jet-expansion. The excitation spectrum consisted of two distinct band systems at 467–430 and 430–405 nm differing significantly in intensity, which the authors assigned to the *S*_1_(*B*_3u_) ← *S*_0_ and *S*_2_(*B*_2u_) ← *S*_0_ transitions of C_32_H_14_ with their 0_0_^0^ bands at 21449 ± 10 (466.2 nm) and ∼23250 cm^–1^ (∼430 nm), respectively. Vibrational analysis of the *S*_1_(*B*_3u_) excitation spectrum revealed short progressions of two normal modes (544 and 793 cm^–1^); due to the larger line widths and band density in the *S*_2_ excitation spectrum, an analogous analysis for *S*_2_ was not attempted. The dispersed fluorescence spectrum covering the range 465–550 nm consisted of sharp peaks of similar progressions to those of the excited state superimposed on a broad background; no direct emission from the *S*_2_ state was observed. Time-resolved fluorescence decay curves could be well-represented by a monoexponential decay and pure radiative lifetimes of ∼2 μs and ∼9 ns were inferred for emission from *S*_1_(*B*_3u_) and *S*_2_(*B*_2u_), respectively. Please note that, after our reassignments to be discussed later, the *S*_1_(*B*_3u_) state in this paragraph should be *S*_2_(*B*_3u_) state, and the *S*_2_(*B*_2u_) state in this paragraph should be *S*_3_(*B*_1g_).

We deposited a mixture of ovalene and *para*-H_2_ onto a substrate at 3.2 K. Upon excitation of this matrix at 22660 cm^–1^ (431.1 nm), we observed the emission system depicted in Figure 2a; the weak first band at 21050 ± 3 cm^–1^ (∼475.1 nm), assigned to the 0_0_^0^ band by comparison with the excitation spectrum, is accompanied by intense features 315, 884, 1129, and 1337 cm^–1^ to lower energies. According to TD-B3LYP-GD3BJ/6-311++G(2d,2p) calculations, the lowest electronic excited singlet state (*S*_1_) is of *B*_2u_ symmetry and has a vertical excitation energy of 20124 cm^–1^ (494.71 nm) and an oscillator strength of 0.18. The *S*_2_ state of *B*_3u_ symmetry is predicted at 21769 cm^–1^ (459.37 nm), 1645 cm^–1^ above *S*_1_(*B*_2u_), with a very small electronic transition dipole strength (0.0004 au), resulting in a negligible predicted oscillator strength. Vertical excitation wavelengths, oscillator strength, and estimated radiative lifetimes of the six lowest excited singlet states of C_32_H_14_ are summarized in Table S1. To assign the obtained spectrum, we simulated the vibrationally resolved electronic emission spectra associated with the *S*_n_–*S*_0_ (*n* = 1–6) transitions by a Franck–Condon Herzberg–Teller approach according to the optimized geometries of the ground and the excited states and their scaled harmonic vibrational frequencies calculated at the (TD-)B3LYP-GD3BJ/6-311++G(2d,2p) level of theory, and compared them with the experimental result in Figure S1. All vibrational frequencies were scaled by 0.98 and those of the seven singlet states (*S*_0_–*S*_6_) involved are listed in Table S2; the scaling factor was derived as outlined in ref (12) from a linear least-squares fit to vibrational frequencies determined from experimental IR spectra of several PAH isolated in solid *para*-H_2_ plotted as a function of vibrational frequencies calculated at this level of theory, as depicted in Figure S2. In Figure S1, spectra associated with the symmetry-allowed transitions from *S*_1_(*B*_2u_) and *S*_6_(*B*_3u_) consist of very intense 0_0_^0^ bands and a few weak vibrational features associated with vibrational normal modes of *a*_1g_ symmetry. However, the most intense feature in the *S*_2_(*B*_3u_) → *S*_0_(*A*_g_) emission spectrum is not the 0_0_^0^ band but instead corresponds to ν_40_ (*b*_1g_, 875 cm^–1^); the 0_0_^0^ band is weak with an intensity <15% relative to the most intense one. Spectra predicted for the symmetry forbidden transitions from excited states of *B*_1g_ symmetry, i.e. *S*_3_(*B*_1g_) and *S*_4_(*B*_1g_), exhibit no origin band and consist of a more intense vibrational structure 1200–2000 cm^–1^ from the 0_0_^0^ bands. No 0_0_^0^ band is predicted for the symmetry forbidden *S*_5_(*A*_g_) → *S*_0_(*A*_g_) transition; this spectrum is mainly composed of three *b*_3u_ symmetric vibrational normal modes ν_116_, ν_120_, and ν_131_. The predicted spectra are consistent with selection rules derived from group theory; our calculations predict that planarity is conserved upon excitation to these six electronic excited states, hence, explaining the contribution of only in-plane vibrations in the emission spectra.

**Figure 2:**
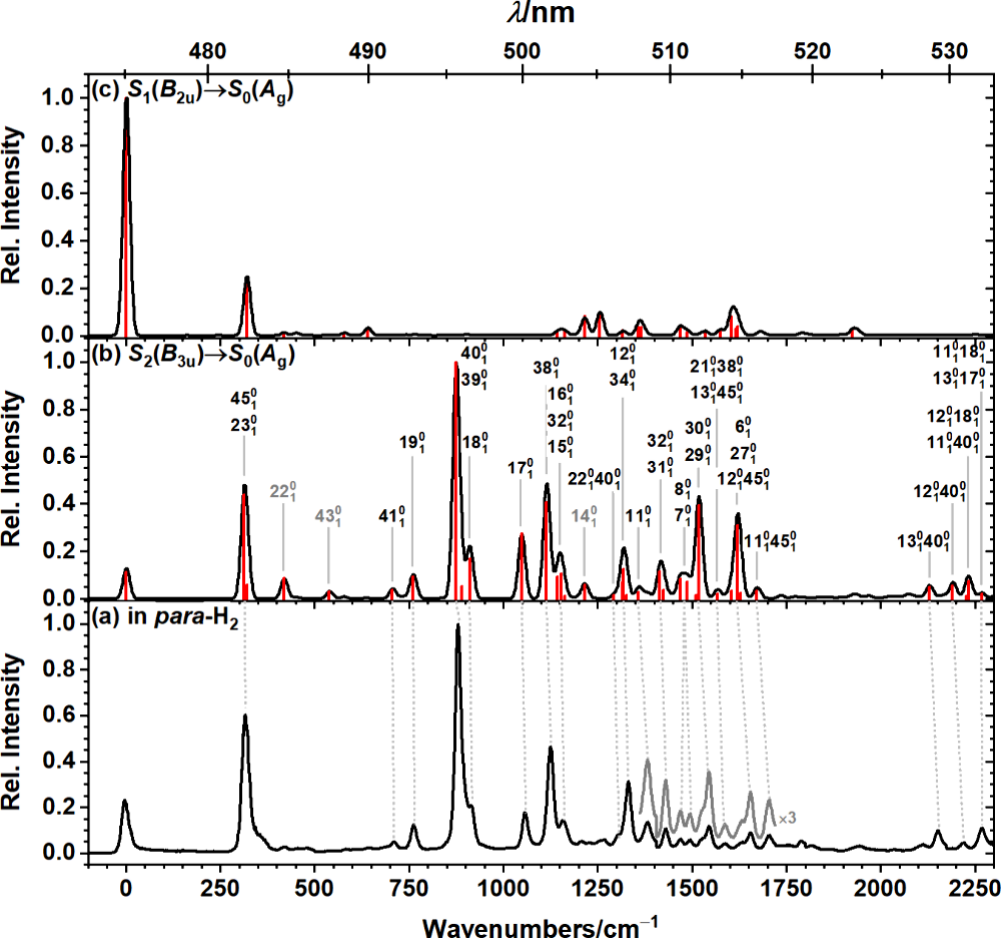
Comparison of observed and simulated fluorescence emission spectra. (a) Dispersed fluorescence spectrum of C_32_H_14_ isolated in solid *para*-H_2_ upon excitation at 22660 cm^–1^ (431.1 nm). The bottom axis indicates the displacement from the origin, observed at 21050 ± 3 cm^–1^ (∼475.1 nm), whereas the top axis indicates the wavelength of the observed spectrum. Simulated (b) *S*_2_(*B*_3u_) → *S*_0_(*A*_g_) and (c) *S*_1_(*B*_2u_) → *S*_0_(*A*_g_) emission spectra (black) obtained by convoluting the Franck–Condon Herzberg–Teller stick spectra (red) with a Gaussian line shape of fwhm 20 cm^–1^. Vibrational frequencies were scaled by 0.98. Gray labeled transitions were not positively identified in the experimental spectrum.

The experimentally observed spectrum is compared with simulated emission spectra of *S*_2_(*B*_3u_) → *S*_0_(*A*_g_) and *S*_1_(*B*_2u_) → *S*_0_(*A*_g_) in Figure 2. With its weak 0_0_^0^ band, dense peak structure between 750 and 1500 cm^–1^, and its most intense peak located at ∼885 cm^–1^, the dispersed fluorescence spectrum of C_32_H_14_ isolated in solid *para*-H_2_ is inconsistent with the simulated *S*_1_(*B*_2u_) → *S*_0_(*A*_g_) emission spectrum, Figure 2c, but instead more closely resembles the simulated *S*_2_(*B*_3u_) → *S*_0_(*A*_g_) emission spectrum, Figure 2b. From time-resolved experiments, we inferred a fluorescence lifetime of ∼1.7 μs, as depicted in Figure S3, in good agreement with the results of Amirav et al.^29^ for jet-cooled C_32_H_14_: for excitation in the range 428.47–466.22 nm, these authors obtained lifetimes of 1.7–2.4 μs. Following the approach outlined in Supporting Information Section SA, we estimated radiative lifetimes of ∼15 ns and ∼1320 ns for emission from the *S*_1_(*B*_2u_) and the *S*_2_(*B*_3u_) states, respectively. According to the comparison of observed spectral pattern and lifetime with theoretical predictions, we assign the observed progression originating at 21050 ± 3 cm^–1^ to emission from the *S*_2_(*B*_3u_) state of C_32_H_14_.

To observe emission from the *S*_2_ state rather than the *S*_1_ state for a molecule isolated in a matrix is a little unusual, if one considers the rapid internal conversion typically observed in matrices. It is known that TD-DFT calculations predict an inverse order for the *S*_1_ and *S*_2_ states of naphthalene and pyrene, both belonging to point group *D*_2h_; this is also the case for the TD-B3LYP-GD3BJ/6-311++G(2d,2p) method employed in this work. Comparing the results of TD-DFT and CC2 calculations for the *S*_1_ and *S*_2_ states of pyrene and three acenes: naphthalene, anthracene, and pentacene, Benkyi et al.^31^ found an inversed order of excited states *S*_1_ and *S*_2_ in the TD-DFT calculations for only naphthalene and pyrene; for the larger acenes, TD-DFT results were consistent with the results from CC2 calculations, which agreed well with previously reported experimental data. Whether our TD-DFT calculations predict the correct order of the *S*_1_ and *S*_2_ states of ovalene cannot be definitely concluded on the basis of the results presented here alone; either higher-level calculations or the experimental confirmation of the existence of a lower-lying electronic excited (*S*_1_) state would be required to confirm the order of these two states. Nevertheless, our results indicate that the excited state should be a *B*_3u_ state rather than a *B*_2u_ state.

Assignments for individual peaks derived from a comparison to the computed stick-spectrum are listed in Table 1 and indicated in Figure 2. Peak positions in the experimental and the convoluted simulated spectrum agree reasonably well with an average absolute deviation of 12 ± 10 cm^–1^. C_32_H_14_ is a planar, *peri*-condensed PAH of *D*_2h_ symmetry with in total 132 vibrational normal modes represented by Γ_vib_ = 23*a*_g_+22*b*_1g_+9*b*_2g_+12*b*_3g_+10*a*_u_+12*b*_1u_+22*b*_2u_+22*b*_3u_. According to group theory, vibrational modes of *a*_g_, *b*_1g_, and *b*_2g_ symmetry can contribute to the *S*_2_(*B*_3u_)–*S*_0_(*A*_g_) transition; however, as *b*_2g_ vibrational modes correspond to out-of-plane deformations of the molecule, their Franck–Condon factors are negligible, and the spectrum is solely composed of in-plane vibrations of *a*_g_ and *b*_1g_ symmetry. Selection rules for IR spectroscopy are disjunct–only *b*_1u_, *b*_2u_, and *b*_3u_ vibrations carry intensity–therefore, our dispersed fluorescence spectrum does not correspond to the IR spectrum of C_32_H_14_ isolated in solid *para*-H_2_ reported by Tsuge et al.^32^ Vibrational quenching in matrix isolated molecules is usually fast, and, in consequence, emission typically occurs from the vibrational ground state of an electronic excited state independent of the vibrational levels excited. As this is not necessarily the case in gas-phase experiments, and, hence, even upon excitation to the same vibrational level, the emitting states for species in the gaseous phase and in solid *para-*H_2_ might differ, resulting in different Franck–Condon factors. Consequently, we refrain from a detailed comparison of our dispersed fluorescence spectrum of C_32_H_14_ isolated in solid *para*-H_2_ to the dispersed fluorescence spectrum of jet-cooled C_32_H_14_ reported by Amirav et al.^29^

**Table 1:** Assignments for Bands Observed in the Dispersed Fluorescence Spectrum of C_32_H_14_ Isolated in Solid *para*-H_2_ and Comparison to Peak Positions in the Simulated *S*_2_(*B*_3u_) → *S*_0_(*A*_g_) Emission Spectrum

*para*-H_2_	B3LYP-GD3BJ				
LIF^a^/cm^–1^	FCHT^b^/cm^–1^	int.^c^/%	scaled^d^/cm^–1^	assign	sym
316	315	43.9	313	45_1_^0^	*b*_1g_
		6.0	320	23_1_^0^	*a*_g_
	(416)	8.7	419	22_1_^0^	*a*_g_
	(537)	3.2	539	43_1_^0^	*b*_1g_
710	706	3.8	706	41_1_^0^	*b*_1g_
765	759	9.3	759	19_1_^0^	*a*_g_
883	875	100.0	876	40_1_^0^	*b*_1g_
		5.2	891	39_1_^0^	*b*_1g_
916^e^	910	17.0	911	18_1_^0^	*a*_g_
1059	1051	27.7	1048	17_1_^0^	*a*_g_
1126	1114	40.7	1114	38_1_^0^	*b*_1g_
1160	1151	9.4	1144	16_1_^0^	*a*_g_
		10.7	1153	37_1_^0^	*b*_1g_
		1.3	1163	15_1_^0^	*a*_g_
	(1213)	5.9	1215	14_1_^0^	*a*_g_
1307^e^	1291	1.4	(1294)	22_1_^0^40_1_^0^	*b*_1g_
1330	1317	7.3	1316	12_1_^0^	*a*_g_
		12.6	1318	34_1_^0^	*b*_1g_
1382	1357	3.0	1357	11_1_^0^	*a*_g_
1431	1416	11.7	1410	32_1_^0^	*b*_1g_
		3.7	1419	31_1_^0^	*b*_1g_
1472	1478	8.6	1468	8_1_^0^	*a*_g_
1497		7.1	1487	7_1_^0^	*a*_g_
1542	1516	1.6	1510	30_1_^0^	*b*_1g_
		39.4	1518	29_1_^0^	*b*_1g_
1583	1566	1.2	(1565)	21_1_^0^38_1_^0^	*b*_1g_
		2.3	(1568)	13_1_^0^45_1_^0^	*b*_1g_
1654	1619	3.4	1604	6_1_^0^	*a*_g_
		30.9	1621	27_1_^0^	*b*_1g_
		2.6	(1629)	12_1_^0^45_1_^0^	*b*_1g_
1704	1671	3.4	(1670)	11_1_^0^45_1_^0^	*b*_1g_
2153	2128	5.1	(2130)	13_1_^0^40_1_^0^	*b*_1g_
2218	2189	5.8	(2191)	12_1_^0^40_1_^0^	*b*_1g_
	(2230)	1.3	(2227)	12_1_^0^18_1_^0^	*a*_g_
		7.7	(2232)	11_1_^0^40_1_^0^	*b*_1g_
2269	2266	2.1	(2268)	11_1_^0^18_1_^0^	*a*_g_
		1.3	(2302)	13_1_^0^17_1_^0^	*a*_g_

a Peak positions relative to the 0_0_^0^ band at 21050 cm^–1^.

b Peak positions from the convoluted simulated stick spectrum calculated at the B3LYP-GD3BJ/6-311++G(2d,2p) level. Vibrational wavenumbers were scaled by 0.98. Bands in parentheses were not positively identified in experiments.

c Intensities relative to the most intense band at 875 cm^–1^ in %; only those greater than 1.5% are listed.

d Harmonic vibrational wavenumbers calculated at the B3LYP-GD3BJ/6-311++G(2d,2p) level and scaled by 0.98. Values for combination bands given in parentheses are the sum of the fundamentals.

e Observed as a shoulder.

Probing fluorescence emission in the range 20114–20281 cm^–1^, corresponding to the most intense peak in the *S*_2_(*B*_3u_) → *S*_0_(*A*_g_) emission spectrum, we obtained the fluorescence excitation spectrum depicted in Figure 3a; assignments derived from a comparison to the simulated *S*_2_(*B*_3u_)← *S*_0_(*A*_g_) absorption spectrum are listed in Table 2 and indicated in Figure 3b. Similar to the dispersed fluorescence spectrum, the excitation spectrum also consists of vibrational normal modes solely of *a*_g_ and *b*_1g_ symmetry. Peak positions inferred from the experimental and simulated spectrum are in satisfactory agreement with an average absolute deviation of 9 ± 7 cm^–1^. The simulated *S*_1_(*B*_2u_) ← *S*_0_(*A*_g_) absorption spectrum is also shown in Figure 3c for comparison; it agrees poorly with our experiments.

**Figure 3:**
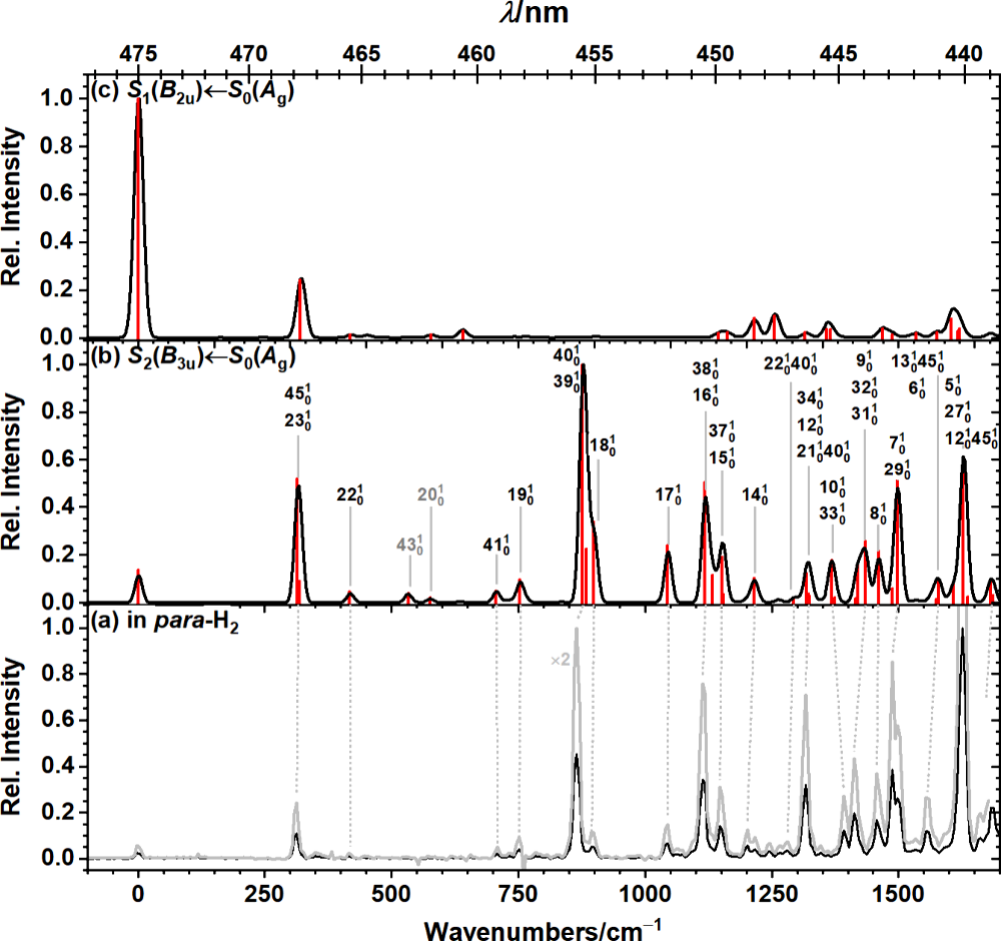
Comparison of observed and simulated fluorescence excitation spectra. (a) Fluorescence excitation spectrum of C_32_H_14_ isolated in solid *para*-H_2_ obtained on probing fluorescence emission in region 20114–20281 cm^–1^. The bottom axis indicates the displacement from the origin, observed at 21050 ± 3 cm^–1^ (∼475.1 nm), whereas the top axis indicates the wavelength of the observed spectrum. The spectrum expanded by a factor of 2 is shown in gray. Simulated (b) *S*_2_(*B*_3u_) ← *S*_0_(*A*_g_) and (c) *S*_1_(*B*_2u_) ← *S*_0_(*A*_g_) absorption spectra (black) obtained by convoluting the Franck–Condon Herzberg–Teller stick spectra (red) with a Gaussian line shape of fwhm 15 cm^–1^.Vibrational frequencies were scaled by 0.98. Peaks labeled in gray were not observed experimentally.

**Table 2:** Assignments for Bands Observed in the Fluorescence Excitation Spectrum of C_32_H_14_ Isolated in Solid *para*-H_2_ and Comparison to Peak Positions in the Simulated *S*_2_(*B*_3u_) ← *S*_0_(*A*_g_) Absorption Spectrum

*para*-H_2_ LIF^a^		B3LYP-GD3BJ			jet-cooled			
nm	cm^–1^	FCHT^b^/cm^–1^	int^c^/%	scaled/cm^–1^	rel^d^/cm^–1^	abs^e^/nm	assign	sym
475.1	0	0	13.3				0_0_^0^	
468.1	313	317	51.9	314	(317)	466.22	45_0_^1^	*b*_1g_
			9.3	319			23_0_^1^	*a*_g_
465.8	418	419	4.6	417			22_0_^1^	*a*_g_
		(534)	4.1	533			43_0_^1^	*b*_1g_
		(578)	2.1	576			20_0_^1^	*a*_g_
459.6	708	708	5.1	707			41_0_^1^	*b*_1g_
458.7	751	755	9.8	753			19_0_^1^	*a*_g_
456.3	865	878	100.0	876	(861)	454.72	40_0_^1^	*b*_1g_
			22.5	884			39_0_^1^	*b*_1g_
455.7	894	899^e^	33.9	899			18_0_^1^	*a*_g_
452.6	1045	1046	24.2	1044			17_0_^1^	*a*_g_
451.2	1113	1119	50.4	1117	(1110)	449.62	38_0_^1^	*b*_1g_
			11.5	1133			16_0_^1^	*a*_g_
450.5	1148	1151	19.1	1151			37_0_^1^	*b*_1g_
			3.6	1155			15_0_^1^	*a*_g_
449.4	1203	1216	10.2	1215			14_0_^1^	*a*_g_
447.8	1281	1294	2.3	1293			22_0_^1^40_0_^1^	*b*_1g_
447.1	1316	1322	12.3	1318	(1303)	445.75	34_0_^1^	*b*_1g_
			3.7	1323			12_0_^1^	*a*_g_
			2.7	1324			21_0_^1^40_0_^1^	*b*_1g_
445.6	1392	1369	17.9	1368	(1376)	444.30	10_0_^1^	*a*_g_
			2.2	1374			33_0_^1^	*b*_1g_
445.2	1412	1434	2.0	1415	(1391)	444.00	9_0_^1^	*a*_g_
			14.7	1420			32_0_^1^	*b*_1g_
			25.7	1434			31_0_^1^	*b*_1g_
444.3	1457	1460	21.7	1461	(1468)	442.50	8_0_^1^	*a*_g_
443.7	1488	1498	5.9	1487	(1480)	442.25	7_0_^1^	*a*_g_
			57.7	1498			29_0_^1^	*b*_1g_
442.3	1559	1578	1.7	1575			13_0_^1^45_0_^1^	*b*_1g_
			9.3	1579			6_0_^1^	*a*_g_
441.0	1626	1628	7.9	1609	(1606)	439.80	5_0_^1^	*a*_g_
			53.7	1628	(1630)	439.35	27_0_^1^	*b*_1g_
			2.6	1637			12_0_^1^45_0_^1^	*b*_1g_
439.8	1688	1684	9.5	1682			10_0_^1^45_0_^1^	*b*_1g_
			3.1	1687			10_0_^1^23_0_^1^	*a*_g_

a Peak positions in wavenumbers relative to the 0_0_^0^ band at 21050 cm^–1^.

b Peak positions from the convoluted simulated stick spectrum calculated at the B3LYP-GD3BJ/6-311++G(2d,2p) level. Vibrational wavenumbers were scaled by 0.98. Bands in parentheses were not observed in experiments.

c Intensities relative to the most intense band at 875 cm^–1^ in %; only those greater than 1.5% are listed.

d Calculated from the wavelengths reported by Amirav et al.^29^ assuming a corrected origin band location of ∼21130 cm^–1^; see text.

e Wavelengths reported by Amirav et al.^29^

Amirav et al.^29,30^ reported the fluorescence excitation spectrum of jet-cooled C_32_H_14_ and provided tentative assignments for the observed spectral features. They assigned the lowest energy band in their spectra, located at 466.22 nm (21449 cm^–1^), to the origin of the transition; this assignment is ∼400 cm^–1^ larger than the origin determined in this work (21050 cm^–1^), significantly larger than our previously reported matrix shifts for the *S*_1_–*S*_0_ transitions of sumanene (C_21_H_12_, ∼55 cm^–1^)^11^ and *peri*-HBC (C_42_H_18_, ∼110 cm^–1^),^13^ and the *D*_1_–*D*_0_ transition of the 1-hydronaphthyl radical (1-C_10_H_9_, 68 cm^–1^).^12^ A peak-by-peak comparison of the excitation spectra reported by Amirav et al.^29,30^ with ours indicates good correlations of reported bands with a matrix red-shift of ∼85 cm^–1^, as shown in Figure S4, but a misassigned origin in Amirav et al.^29,30^ According to our simulations and experimental results, the 0_0_^0^ band is about a factor of 4 weaker in intensity than the first vibrational band, corresponding to a superposition of ν_45_ (314 cm^–1^, *b*_1g_) and ν_23_ (319 cm^–1^, *a*_g_) predicted at 317 cm^–1^. The 0_0_^0^ band reported by Amirav et al.^29,30^ is hence misassigned; assigning this band to the superposition of ν_45_ and ν_23_ instead, significantly improves the agreement of their reported spectrum with our simulated *S*_2_(*B*_3u_) ← *S*_0_(*A*_g_) absorption spectrum, reducing the average absolute deviation to 9 ± 7 cm^–1^, comparable to our experimental data. Wavenumbers for the spectrum of jet-cooled C_32_H_14_ relative to the corrected transition origin are listed in Table 2 for comparison. A comparison to the absorption spectrum of C_32_H_14_ isolated in solid Ne is provided in Table S3.

In line with the spectra of jet-cooled C_32_H_14_ reported by Amirav et al.,^29,30^ and of C_32_H_14_ isolated in solid Ne reported by Ehrenfreund et al.^15^ and Ruiterkamp et al.,^27^ our fluorescence excitation spectrum of C_32_H_14_ isolated in solid *para*-H_2_ also exhibits enhanced peak intensities for bands >1600 cm^–1^. This increase is not predicted by the simulated *S*_2_(*B*_3u_) ← *S*_0_(*A*_g_) absorption spectrum and, therefore, likely indicates contributions from higher electronically excited states. A tentative assignment of the additional features to the *S*_3_(*B*_1g_) ← *S*_0_(*A*_g_) transition is discussed in Supporting Information Section SB. An extended fluorescence excitation spectrum of C_32_H_14_ isolated in solid *para*-H_2_ is depicted in Figure S5 and compared to simulated absorption spectra of the *S*_2_(*B*_3u_)–*S*_0_(*A*_g_), *S*_3_(*B*_1g_)–*S*_0_(*A*_g_), and *S*_4_(*B*_1g_)–*S*_0_(*A*_g_) transitions. According to this assignment, the 0_0_^0^ band of the *S*_3_(*B*_1g_)–*S*_0_(*A*_g_) transition should be located near 21400 cm^–1^, ∼350 cm^–1^ higher in energy than the 0_0_^0^ band of the *S*_2_(*B*_3u_)–*S*_0_(*A*_g_) transition. Indeed, a very weak feature located at 21405 ± 5 cm^–1^ was observed in our fluorescence excitation spectrum shown in Figure S6. The observed wavenumbers and tentative assignments are listed in Table S4. The *S*_3_(*B*_1g_)–*S*_2_(*B*_3u_) energy gap of ∼355 cm^–1^, is consistent, although somewhat smaller, with the energy gap (450 ± 50 cm^–1^) inferred by Kropp and Stantley^24^ from their temperature-dependent measurements of anomalous fluorescence of C_32_H_14_ in organic solvents, but differs significantly from the *S*_3_(*B*_1g_)– *S*_2_(*B*_3u_) energy gap (vertical transition) of ∼3525 cm^–1^ predicted by our TD-DFT calculations. Even when we take the 1639 cm^–1^ difference between the origin and the most intense band in the *S*_3_(*B*_1g_) state into account, the estimated (local minimum) energy gap of 1886 cm^–1^ is still greater than the experimental value. However, this deviation is within a root-mean square error of 0.467 eV (∼3750 cm^–1^) for vertical excitation energies of singlet transitions calculated with the B3LYP functional reported by Liang et al.^33^

Jenniskens and Désert^34^ identified only one DIB near 435 nm; this DIB at 4176.47 Å, however, is located ∼900 cm^–1^ higher in energy then the most intense peak in the fluorescence excitation spectrum presented here at 434.4 nm (Figure S5), tentatively assigned to the *S*_3_(*B*_1g_) ← *S*_0_(*A*_g_) transition, mode ν_93_. A contribution of C_32_H_14_ to the DIB is therefore unlikely, in line with the conclusions previously drawn by Ehrenfreund et al.^15^ and Ruiterkamp et al.^27^

In summary, we propose a revision of the transition origin and the upper electronic-state assignment for the fluorescence excitation spectrum of jet-cooled ovalene in the region 430–467 nm previously reported by Amirav et al.^29,30^ We assigned the transition origin to be 21050 ± 3 cm^–1^ for C_32_H_14_ isolated in solid *para*-H_2_ according to our dispersed fluorescence and fluorescence excitation spectra and this transition should be assigned to the *S*_2_(*B*_3u_)–*S*_0_(*A*_g_) rather than the *S*_1_– *S*_0_ transition. From the revised 0_0_^0^ position we inferred a *para*-H_2_ red matrix shift of ∼85 cm^–1^ relative to the gas-phase value. The fluorescence lifetime of ∼1.7 μs in solid *para*-H_2_ is consistent with the lifetime of jet-cooled C_32_H_14_ reported by Amirav et al.^29,30^ and a radiative emission lifetime of 1.3 μs estimated for the *S*_2_(*B*_3u_)–*S*_0_(*A*_g_) transition on the basis of TD-DFT calculations. For the *S*_1_(*B*_2u_)–*S*_0_(*A*_g_) transition, our calculations indicate a significantly shorter radiative emission lifetime of ∼14 ns and a Franck–Condon envelop significantly distinct from our observations. We provided detailed vibrational assignments for vibrational features in the dispersed fluorescence and fluorescence excitation spectra to vibrational normal modes of *b*_1g_ and *a*_g_ symmetry by comparison of our experimental data to simulated absorption and emission spectra. Additional features >1600 cm^–1^ above the *S*_2_(*B*_3u_)– *S*_0_(*A*_g_) transition origin are tentatively assigned to the *S*_3_(*B*_1g_)– *S*_0_(*A*_g_) system, implying a transition origin ∼21405 cm^–1^; the *S*_3_(*B*_1g_) state is ∼355 cm^–1^ above the *S*_2_(*B*_3u_) state. The actual location of the *S*_1_(*B*_2u_) state requires further investigations.

## Methods

The *para*-H_2_/LIF experiment has been described in detail previously,^11−13^ therefore, only a brief outline is given here. A nickel-coated copper plate, mounted onto the second stage of a closed-cycle helium refrigerator and cooled to ∼3 K, served as a matrix substrate and the reflective surface for absorption. Matrix spectra were measured with a Fourier-Transform infrared (FTIR) spectrometer (Bruker, iFS66v) equipped with a KBr beam splitter and a Hg–Cd–Te detector cooled with liquid N_2_. Spectra, typically covering 500–5000 cm^–1^ at resolution 0.25 cm^–1^, were acquired over 300 scans.

To record dispersed fluorescence spectra, the matrix was irradiated with the output of an optical parametric oscillator (OPO, EKSPLA NT340) operated at 10 Hz and pumped by a frequency-tripled Nd:YAG laser (EKSPLA, NT300) at 355 nm. The OPO output beam was expanded to a diameter of ∼1.5 cm to maximize the excitation area on the matrix. Emitted light was collected with a convex lens (*f* = 50 mm), collimated, and transmitted to the spectrograph through an optical fiber. The spectrograph consists of a monochromator (Andor Shamrock SR500i), with a focal length of 0.5 m and a holographic grating (2400 grooves mm^–1^, reciprocal linear dispersion 0.83 nm mm^–1^), and an intensified charge coupled device (iCCD, Andor iStar DH320T-18U-73, 1024 × 225 pixels, pixel size 26 μm × 26 μm). In this configuration, each pixel of the iCCD corresponds to 0.022 nm, which is ≤1 cm^–1^ for wavelength ≥470 nm. All dispersed fluorescence spectra have been corrected for wavelength dependent changes in sensitivity of the grating and the detector; absolute wavelengths were calibrated with a Hg(Ar) lamp.

Fluorescence excitation spectra were recorded by probing fluorescence emission in a specific wavelength range while stepping the OPO output wavelength in increments of 0.1 nm; in the range 410–480 nm, the spectral line width of the OPO was 4 cm^–1^. To maximize the detected fluorescence signal, another grating (600 grooves mm^–1^, blaze 500 nm) was employed and the entrance slit of the monochromator was set to ∼2.5 mm.

All quantum-chemical calculations were performed with the Gaussian 16 program package, Revision A.03.^35^ Geometry optimizations and harmonic-frequency calculations for the electronic ground state and selected electronic excited states of ovalene were performed with the (time-dependent) density functional theory, (TD-)B3LYP-GD3BJ/6-311++G(2d,2p) method, including Grimme’s D3 dispersion correction with Becke-Johnson damping (GD3BJ)^36^ to account for electrostatic interactions. Vibronic absorption and emission spectra were simulated according to the optimized geometries and scaled harmonic vibrational frequencies of the electronic states involved by the Franck–Condon Herzberg–Teller approach. For comparison to our experimental results, we convoluted the computed stick-spectra with a Gaussian line shape using a full-width-half-maximum (fwhm) of 20 and 15 cm^–1^ for the simulated emission and absorption spectra, respectively; these parameters were chosen to match the line shapes observed in our experiments. Hamonic vibrational frequencies were scaled by empirical scaling factors of 0.98 to account for systematic deviations due to calculation errors and effects of the matrix environment

## Supporting Information Available

Estimation of fluorescence lifetimes, tentative assignment of the *S*_3_(*B*_1g_) ← *S*_0_(*A*_g_) excitation spectrum, predicted properties of the first six excited states of C_32_H_14_, calculated vibrational wavenumbers of the ground and excited states of C_32_H_14_, comparison of peak positions in the *S*_2_(*B*_3u_) ← *S*_0_(*A*_g_) spectrum with literature, tentative assignments of the observed *S*_3_(*B*_1g_) ← *S*_0_(*A*_g_) excitation spectrum, simulated emission spectra for the six lowest-energy transitions of C_32_H_14_, determination of the vibrational scaling factor, fluorescence lifetime measurement, comparison of spectra of jet-cooled C_32_H_14_ with C_32_H_14_ in solid *para*-H_2_, comparison of extended fluorescence excitation spectrum of C_32_H_14_ isolated in solid *para*-H_2_ with simulations, and tentative assignment of the *S*_3_(*B*_1g_)–*S*_0_(*A*_g_) 0_0_^0^ band of C_32_H_14_ (PDF)
Transparent Peer Review report available (PDF)
