# Supplementary material for: Electronic Spectroscopy of Ovalene: Reassignment of the S2(B3u)– S0(Ag) Transition
Source: J Phys Chem Lett. 2024 Oct 16;15(42):10696–702. doi: 10.1021/acs.jpclett.4c02494 (PMC11514017; doi:10.1021/acs.jpclett.4c02494)
Supplement: Supplementary file 1 — jz4c02494_si_001.pdf [file jz4c02494_si_001.pdf]

Supporting Information

Electronic Spectroscopy of Ovalene: Reassignment of the $S_2(B_{3u})- S_0(A_g)$ Transition

Isabelle Weber,[†]* *Johanna Langner*,[†] *Henryk A. Witek*,[†] *and Yuan-Pern Lee*[†,‡,*]

[†] Department of Applied Chemistry and Institute of Molecular Science, National Yang Ming Chiao Tung University, Hsinchu 3000093, Taiwan

[‡] Center for Emergent Functional Matter Science, National Yang Ming Chiao Tung University, Hsinchu 300093, Taiwan

Table of Contents

Section SA. Estimation of fluorescence lifetimes. S2

Section SB. Tentative assignment of the $S_3(B_{1g})$←$S_0(A_g)$ excitation spectrum......................S2

Table S1. Vertical Excitation Wavelengths (λ) and Energies (ΔE), Oscillator Strengths (f), and Estimated Lifetimes (τ) of the Six Lowest Singlet Excited States of $C_{32}H_{14}$ as Predicted with the TD-B3LYP-G3BJ/6-311++G(2d,2p) Method S5

Table S2. Scaled Harmonic Vibrational Wavenumbers of the Ground and the Six Lowest Excited States of $C_{32}H_{14}$ Calculated at the B3LYP-GD3BJ/6-311++G(2d,2p) Level. S6

Table S3. Comparison of Peak Positions in the $S_2(B_{3u})$←$S_0(A_g)$ Fluorescence Excitation Spectrum of $C_{32}H_{14}$ Isolated in Solid *para*-H_2 to Previous Work S9

Table S4. Tentative Assignments for Bands Observed in the $S_3(B_{1g})$←$S_0(A_g)$ Fluorescence Excitation Spectrum of $C_{32}H_{14}$ Isolated in Solid *para*-H_2 and Comparison to Peak Positions in the Simulated Absorption Spectrum and Previous Experimental Work...........................S10

Figure S1. Comparison of experimental dispersed fluorescence spectrum with vibrationally resolved S_n←$S_0(A_g)$ (n = 1−6) electronic absorption spectra S11

Figure S2. Linear least-squares fit of experimental vibrational wavenumbers vs. calculated harmonic vibrational wavenumbers S12

Figure S3. Time-resolved integrated fluorescence emission (black dots) of $C_{32}H_{14}$ isolated in solid *para*-H_2 upon excitation at 22119 cm^{-1} (452.1 nm) S13

Figure S4. Comparison of observed $S_2(B_{3u})$←$S_0(A_g)$ fluorescence excitation spectrum of $C_{32}H_{14}$ isolated in solid *para*-H_2 with the spectrum of jet-cooled $C_{32}H_{14}$ by Amirav et al. ..S14

Figure S5. Comparison of experimental fluorescence excitation spectrum with simulated spectra of $S_2(B_{3u})$←$S_0(A_g)$, $S_3(B_{1g})$←$S_0(A_g)$, and $S_4(B_{1g})$←$S_0(A_g)$ S15

Figure S6. Partial fluorescence excitation spectrum of $C_{32}H_{14}$ isolated in solid *para*-H_2 indicating the 0_0^0 bands of the $S_2(B_{3u})$←$S_0(A_g)$ and $S_3(B_{1g})$←$S_0(A_g)$ transitions...................S16

References...S17

Section SA. Estimation of fluorescence lifetimes

To estimate radiative lifetimes from the computed excited state properties, we employed a simplified model to calculate the Einstein coefficient for spontaneous emission (A_{ul}) from an upper electronic excited state (u) to a lower electronic excited state (l). The excited state (radiative) lifetimes (τ) are related to A_{ul} through

$$A_{ul} = \frac{1}{\tau}. \quad \text{(S1)}$$

The Einstein coefficient can be expressed as a function of oscillator strength (f):

$$A_{ul} = \frac{2\pi f v_{ul}^2 n^3 e^2}{\epsilon_0 m_e c_0^3}, \quad \text{(S2)}$$

in which v_{ul} is the frequency of the transition between the upper and the lower states, n is the refractive index of the surrounding medium, which is 1.13 for *para*-H_2 at ~4 K,[1] e is the elementary charge, ε_0 is the vacuum permittivity, m_e is the electron mass, and c_0 is the speed of light in vacuo. The oscillator strength f can be expressed as a function of the extinction coefficient ε,[2] and thereby estimated from simulated absorption spectra also for transitions with a predicted oscillator strength of zero:

$$f = 4.32 \times 10^{-9} \int \epsilon(v) dv. \quad \text{(S3)}$$

The integrated extinction coefficient ($\int \epsilon(v) dv$) can be derived from the Franck-Condon Herzberg-Teller simulations.

Section SB. Tentative assignment of the $S_3(B_{1g})\leftarrow S_0(A_g)$ excitation spectrum

In line with the spectra of jet-cooled $C_{32}H_{14}$ reported by Amirav et al.,[3,4] and of $C_{32}H_{14}$ isolated in solid Ne reported by Ehrenfreund et al.[5] and Ruiterkamp et al.,[6] our fluorescence excitation spectrum of $C_{32}H_{14}$ isolated in solid *para*-H_2 also exhibits enhanced peak intensities for bands >1600 cm^{-1}. This increase is not predicted by the simulated $S_2(B_{3u})\leftarrow S_0(A_g)$ absorption spectrum and, therefore, likely indicates contributions from higher electronically

excited states. An extended fluorescence excitation spectrum of $C_{32}H_{14}$ isolated in solid *para*-H_2 is depicted in Figure S5 and compared to simulated absorption spectra of the $S_2(B_{3u})$–$S_0(A_g)$, $S_3(B_{1g})$–$S_0(A_g)$, and $S_4(B_{1g})$–$S_0(A_g)$ transitions; for a better comparison, the simulated absorption spectra of $S_3(B_{1g})$–$S_0(A_g)$ and $S_4(B_{1g})$–$S_0(A_g)$ were shifted by +351 and +472 cm^{-1}, respectively, to align either the most intense feature (S_3) or the intense doublet structure (S_4) with the most intense peak in the experimental spectrum. The simulated $S_3(B_{1g})\leftarrow S_0(A_g)$ absorption spectrum, with a missing transition origin, consists of an intense peak at 1619 cm^{-1} and three characteristic features at 1431, 1551, and 1934 cm^{-1} with relative intensities of 18, 19, and 29 %, respectively. The separation between the latter band and the most intense feature of 315 cm^{-1} in the simulated spectrum is consistent with the separation between the two intense bands in the observed fluorescence excitation spectrum (~320 cm^{-1}); the weaker features, predicted 68 and 188 cm^{-1} to the red from the most intense feature also match satisfactorily with a shoulder and weak side-band in the experimental spectrum. According to this assignment, the 0_0^0 band of the $S_3(B_{1g})$–$S_0(A_g)$ transition should be located near 21400 cm^{-1}, ~350 cm^{-1} higher in energy than the 0_0^0 band of the $S_2(B_{3u})$–$S_0(A_g)$ transition. Indeed, a very weak feature located at 21405±5 cm^{-1} was observed in our fluorescence excitation spectrum shown in Figure S6. It must be noted, however, that the intense peaks centred at ~22967 and ~23295 cm^{-1} in our fluorescence excitation spectrum are split into two components separated by ~50 cm^{-1}. The simulated $S_4(B_{1g})\leftarrow S_0(A_g)$ absorption spectrum with two pairs of doublets with a splitting of ~113 cm^{-1} located at 1384 and 1701 cm^{-1}, respectively, qualitatively resembles the structure of the experimental spectrum, however, actual peak positions do not match satisfactorily.

We therefore tentatively assign these additional bands to absorption to the $S_3(B_{1g})$ state of $C_{32}H_{14}$. The observed wavenumbers and tentative assignments are listed in Table S4. The $S_3(B_{1g})$–$S_2(B_{3u})$ energy gap of ~355 cm^{-1}, is consistent, although somewhat smaller, with the

energy gap (450±50 cm^{-1}) inferred by Kropp and Stantley[7] from their temperature-dependent measurements of anomalous fluorescence of $C_{32}H_{14}$ in organic solvents, but differs significantly from the $S_3(B_{1g})$–$S_2(B_{3u})$ energy gap (in vertical transition from the ground electronic state) of ~3525 cm^{-1} predicted by our TD-DFT calculations. Even when we take the 1639 cm^{-1} difference between the origin and the most intense band in the $S_3(B_{1g})$ state into account, the estimated energy gap of 1886 cm^{-1} is still greater than the experimental value. In a recent benchmark study of TD-DFT vertical excitation energies computed with various functionals in combination with the aug-cc-PVTZ basis set, Liang et al.[8] reported a root-mean square error of 0.467 eV (~3750 cm^{-1}) for vertical excitation energies of singlet transitions calculated with the B3LYP functional; the deviation in the $S_3(B_{1g})$ state is within this estimated error. The estimated $S_3(B_{1g})$ lifetime in Table S1, 230 ns, is much shorter than the lifetime of the $S_2(B_{3u})$ state (estimated to be 1320 ns and observed to be ~1700 ns in solid *para*-H_2). We found identical lifetimes upon excitation of $C_{32}H_{14}$ in solid *para*-H_2 to $S_3(B_{1g})$ and $S_2(B_{3u})$, consistent with the expectation that the emission from $S_2(B_{3u})$ is the rate-determining step in the relaxation cascade.

Table S1. Vertical Excitation Wavelengths (λ) and Energies (ΔE), Oscillator Strengths (f), and Estimated Lifetimes (τ) of the Six Lowest Singlet Excited States of $C_{32}H_{14}$ as Predicted with the TD-B3LYP-G3BJ/6-311++G(2d,2p) Method

state	sym.	λ /nm	ΔE /cm^{-1}	f	τ /ns
S_1	B_{2u}	494.71	20124	0.18	15
S_2	B_{3u}	459.37	21769	~0.00	1320
S_3	B_{1g}	395.35	25294	~0.00	230
S_4	B_{1g}	377.00	26525	~0.00	63
S_5	A_g	359.58	27810	~0.00	32
S_6	B_{3u}	349.53	28610	1.05	1

Table S2. Scaled Harmonic Vibrational Wavenumbers of the Ground and the Six Lowest Excited States of $C_{32}H_{14}$ Calculated at the B3LYP-GD3BJ/6-311++G(2d,2p) Level.[a]

mode	symmetry	S_0	S_1	S_2	S_3	S_4	S_5	S_6
ν_1	a_g	3122	3123	3124	3126	3123	3126	3123
ν_2	a_g	3119	3121	3121	3120	3121	3122	3119
ν_3	a_g	3105	3106	3107	3108	3113	3107	3108
ν_4	a_g	3101	3103	3103	3104	3104	3100	3103
ν_5	a_g	1617	1603	1609	1592	1618	1590	1591
ν_6	a_g	1604	1596	1579	1580	1596	1580	1573
ν_7	a_g	1487	1501	1487	1480	1502	1479	1479
ν_8	a_g	1468	1467	1461	1465	1484	1457	1452
ν_9	a_g	1441	1422	1415	1420	1397	1424	1405
ν_{10}	a_g	1366	1366	1368	1369	1388	1386	1363
ν_{11}	a_g	1357	1356	1362	1355	1358	1361	1356
ν_{12}	a_g	1316	1322	1323	1323	1322	1305	1319
ν_{13}	a_g	1255	1244	1261	1241	1248	1236	1252
ν_{14}	a_g	1215	1227	1215	1227	1238	1211	1210
ν_{15}	a_g	1163	1157	1155	1151	1158	1146	1152
ν_{16}	a_g	1144	1145	1133	1142	1148	1138	1134
ν_{17}	a_g	1048	1049	1044	1047	1055	1033	1042
ν_{18}	a_g	911	896	899	898	893	903	901
ν_{19}	a_g	759	747	753	749	760	750	749
ν_{20}	a_g	578	573	576	569	590	571	573
ν_{21}	a_g	451	447	448	450	458	447	447
ν_{22}	a_g	419	418	417	414	424	414	416
ν_{23}	a_g	320	317	319	316	317	320	319
ν_{24}	b_{1g}	3120	3121	3121	3124	3121	3129	3121
ν_{25}	b_{1g}	3104	3106	3108	3109	3106	3107	3105
ν_{26}	b_{1g}	3102	3103	3104	3103	3103	3105	3103
ν_{27}	b_{1g}	1621	1565	1628	1557	1570	2300	1561
ν_{28}	b_{1g}	1599	1547	1565	1500	1487	1563	1493
ν_{29}	b_{1g}	1518	1499	1498	1473	1475	1496	1475
ν_{30}	b_{1g}	1510	1469	1491	1422	1424	1457	1452
ν_{31}	b_{1g}	1423	1418	1434	1396	1412	1423	1419
ν_{32}	b_{1g}	1414	1393	1420	1392	1398	1376	1395
ν_{33}	b_{1g}	1381	1363	1374	1312	1322	1349	1337
ν_{34}	b_{1g}	1318	1321	1318	1225	1288	1262	1297
ν_{35}	b_{1g}	1227	1213	1226	1196	1210	1218	1224
ν_{36}	b_{1g}	1199	1191	1203	1157	1189	1171	1173
ν_{37}	b_{1g}	1153	1130	1151	1121	1128	1125	1120
ν_{38}	b_{1g}	1114	1114	1117	1059	1110	1101	1112
ν_{39}	b_{1g}	891	893	884	867	884	899	878
ν_{40}	b_{1g}	875	865	876	855	850	839	865
ν_{41}	b_{1g}	706	707	706	699	705	704	703
ν_{42}	b_{1g}	642	642	637	626	636	639	635

ν_{43}	b_{1g}	539	535	533	535	537	530	530
ν_{44}	b_{1g}	475	470	475	464	467	470	471
ν_{45}	b_{1g}	313	309	314	309	289	293	296
ν_{46}	b_{2g}	966	940	947	912	938	933	942
ν_{47}	b_{2g}	847	827	829	832	834	832	828
ν_{48}	b_{2g}	789	777	771	763	778	767	766
ν_{49}	b_{2g}	744	726	729	702	725	720	726
ν_{50}	b_{2g}	567	559	559	568	559	579	556
ν_{51}	b_{2g}	522	500	503	449	488	494	499
ν_{52}	b_{2g}	321	313	310	312	318	326	306
ν_{53}	b_{2g}	189	187	185	171	187	178	184
ν_{54}	b_{2g}	123	116	117	120	116	128	116
ν_{55}	b_{3g}	971	958	951	966	956	946	947
ν_{56}	b_{3g}	952	937	919	919	933	934	920
ν_{57}	b_{3g}	888	865	865	887	842	874	854
ν_{58}	b_{3g}	806	788	790	786	782	787	785
ν_{59}	b_{3g}	773	756	758	748	754	741	751
ν_{60}	b_{3g}	713	695	698	688	700	676	689
ν_{61}	b_{3g}	624	602	614	610	592	570	603
ν_{62}	b_{3g}	556	552	535	555	546	504	532
ν_{63}	b_{3g}	500	472	469	484	472	450	463
ν_{64}	b_{3g}	338	321	320	338	323	329	315
ν_{65}	b_{3g}	265	253	255	249	250	254	256
ν_{66}	b_{3g}	149	147	144	153	143	142	142
ν_{67}	a_u	970	958	949	966	956	945	945
ν_{68}	a_u	951	935	918	907	931	929	919
ν_{69}	a_u	819	808	804	794	814	800	799
ν_{70}	a_u	799	787	785	768	790	762	779
ν_{71}	a_u	657	653	641	643	650	647	636
ν_{72}	a_u	611	597	594	583	567	573	590
ν_{73}	a_u	471	463	446	450	469	461	441
ν_{74}	a_u	328	325	320	313	325	311	317
ν_{75}	a_u	252	245	248	248	240	247	244
ν_{76}	a_u	59	58	57	62	56	57	55
ν_{77}	b_{1u}	968	943	949	921	939	938	944
ν_{78}	b_{1u}	891	870	869	893	851	878	860
ν_{79}	b_{1u}	839	815	818	827	806	825	816
ν_{80}	b_{1u}	789	771	772	757	764	766	765
ν_{81}	b_{1u}	760	746	746	733	746	737	742
ν_{82}	b_{1u}	634	615	622	620	607	620	619
ν_{83}	b_{1u}	542	526	527	519	514	512	523
ν_{84}	b_{1u}	427	403	412	420	395	408	402
ν_{85}	b_{1u}	335	323	320	319	312	313	318
ν_{86}	b_{1u}	208	199	196	212	196	212	194
ν_{87}	b_{1u}	102	99	102	94	100	92	102
ν_{88}	b_{1u}	60	58	58	57	57	60	58

ν_{89}	b_{2u}	3120	3122	3122	3125	3121	3125	3122
ν_{90}	b_{2u}	3105	3107	3108	3109	3112	3107	3108
ν_{91}	b_{2u}	3103	3104	3106	3104	3106	3106	3104
ν_{92}	b_{2u}	3101	3102	3103	3103	3103	3100	3102
ν_{93}	b_{2u}	1630	1593	1576	1619	1564	1565	1731
ν_{94}	b_{2u}	1565	1542	1521	1542	1528	1538	1521
ν_{95}	b_{2u}	1533	1507	1507	1510	1498	1521	1517
ν_{96}	b_{2u}	1454	1435	1447	1438	1441	1445	1447
ν_{97}	b_{2u}	1446	1425	1423	1430	1385	1432	1442
ν_{98}	b_{2u}	1400	1328	1347	1402	1384	1359	1375
ν_{99}	b_{2u}	1313	1293	1300	1300	1292	1295	1301
ν_{100}	b_{2u}	1274	1265	1254	1271	1268	1251	1265
ν_{101}	b_{2u}	1227	1222	1222	1219	1220	1215	1215
ν_{102}	b_{2u}	1163	1149	1152	1141	1149	1149	1149
ν_{103}	b_{2u}	1073	1054	1062	1065	1060	1042	1066
ν_{104}	b_{2u}	918	916	910	917	915	917	909
ν_{105}	b_{2u}	786	779	764	786	774	785	770
ν_{106}	b_{2u}	707	704	701	704	701	703	698
ν_{107}	b_{2u}	657	654	653	653	656	651	653
ν_{108}	b_{2u}	577	573	572	579	570	574	581
ν_{109}	b_{2u}	492	483	481	491	477	489	493
ν_{110}	b_{2u}	276	273	275	272	271	275	274
ν_{111}	b_{3u}	3121	3123	3123	3125	3123	3126	3122
ν_{112}	b_{3u}	3119	3120	3120	3120	3120	3122	3121
ν_{113}	b_{3u}	3103	3104	3104	3108	3104	3106	3105
ν_{114}	b_{3u}	1615	1591	1558	1584	1600	1572	1718
ν_{115}	b_{3u}	1584	1552	1550	1551	1551	1550	1542
ν_{116}	b_{3u}	1495	1470	1455	1493	1486	1518	1526
ν_{117}	b_{3u}	1439	1439	1435	1441	1452	1445	1431
ν_{118}	b_{3u}	1412	1417	1403	1412	1405	1397	1393
ν_{119}	b_{3u}	1382	1374	1373	1387	1377	1380	1369
ν_{120}	b_{3u}	1380	1364	1330	1364	1364	1359	1356
ν_{121}	b_{3u}	1315	1288	1319	1350	1345	1340	1312
ν_{122}	b_{3u}	1242	1228	1237	1224	1229	1229	1234
ν_{123}	b_{3u}	1208	1218	1208	1202	1209	1206	1197
ν_{124}	b_{3u}	1170	1176	1176	1177	1178	1179	1157
ν_{125}	b_{3u}	1135	1143	1131	1136	1148	1139	1134
ν_{126}	b_{3u}	1071	1067	1067	1063	1064	1066	1057
ν_{127}	b_{3u}	978	972	969	974	972	978	962
ν_{128}	b_{3u}	780	769	775	768	773	770	773
ν_{129}	b_{3u}	677	670	669	668	657	671	670
ν_{130}	b_{3u}	537	535	535	532	534	531	539
ν_{131}	b_{3u}	425	417	419	422	419	406	435
ν_{132}	b_{3u}	392	390	388	393	393	388	386

[a]in cm^{-1}. Scaling factor is 0.98; see text.

Table S3. Comparison of Peak Positions in the $S_2(B_{3u})\leftarrow S_0(A_g)$ Fluorescence Excitation Spectrum of $C_{32}H_{14}$ Isolated in Solid *para*-H_2 to Previous Work

para-H_2 /nm	Ne[a] /nm	jet-cooled[b] /nm	assignment[c]	symmetry
475.1			0_0^0	
468.1		466.22	45_0^1	b_{1g}
			23_0^1	a_g
465.8			22_0^1	a_g
			43_0^1	b_{1g}
			20_0^1	a_g
459.6			41_0^1	b_{1g}
458.7			19_0^1	a_g
456.3	455.19	454.72	40_0^1	b_{1g}
			39_0^1	b_{1g}
455.7[d]			18_0^1	a_g
452.6			17_0^1	a_g
451.2	451.52	449.62	38_0^1	b_{1g}
			16_0^1	a_g
450.5	450.11		37_0^1	b_{1g}
			15_0^1	a_g
449.4			14_0^1	a_g
447.8			$22_0^1 40_0^1$	b_{1g}
447.1		445.75	34_0^1	b_{1g}
			12_0^1	a_g
			$21_0^1 40_0^1$	b_{1g}
445.6	446.04	444.30	10_0^1	a_g
			33_0^1	b_{1g}
445.2		444.00	9_0^1	a_g
			32_0^1	b_{1g}
			31_0^1	b_{1g}
444.3	444.29	442.50	8_0^1	a_g
443.7		442.25	7_0^1	a_g
			29_0^1	b_{1g}
442.3	442.58		$13_0^1 45_0^1$	b_{1g}
			6_0^1	a_g
441.0		439.80	5_0^1	a_g
		439.35	27_0^1	b_{1g}
			$12_0^1 45_0^1$	b_{1g}
439.8	439.94		$10_0^1 45_0^1$	b_{1g}
			$10_0^1 23_0^1$	a_g

[a]Peak positions in the absorption spectrum of $C_{32}H_{14}$ isolated in solid Ne reported by Ruiterkamp et al.[6] [b]Peak positions in the fluorescence excitation spectrum of jet-cooled $C_{32}H_{14}$ reported by Amirav et al.[3] [c]Peak assignments derived in this work, c.f. Table 2. [d]Observed as a shoulder to the peak at 445.7 nm.

Table S4. Tentative Assignments for Bands Observed in the $S_3(B_{1g})\leftarrow S_0(A_g)$ Fluorescence Excitation Spectrum of $C_{32}H_{14}$ Isolated in Solid *para*-H_2 and Comparison to Peak Positions in the Simulated Absorption Spectrum and Previous Experimental Work

para-H_2		B3LYP-GD3BJ			Ne[a]	jet-cooled[b]	assign.	sym.
LIF[c]		FCHT[d]	int.[e]	scaled[f]				
/nm	/cm^{-1}	/cm^{-1}	/%	/cm^{-1}	/nm	/nm		
467.2	0	0	0	0			0^0_0	
		(422)	3.3	422			131^1_0	b_{3u}
		(578)	3.4	579			108^1_0	b_{2u}
452.0	718	704	5.4	704			106^1_0	b_{2u}
447.8	926	919	3.0	917			104^1_0	b_{2u}
442.3	1204	1201	1.6	1202			123^1_0	b_{3u}
441.0	1271	1272	2.7	1271			100^1_0	b_{2u}
439.1	1369	1366	1.2	1364			120^1_0	b_{3u}
438.3	1410	1401	2.6	1402			98^1_0	b_{2u}
437.7	1442	1431	18.1	1430		428.15	97^1_0	b_{2u}
435.3	1568	1551	19.0	1551		427.39	115^1_0	b_{3u}
434.3	1619	1619	100.0	1619	430.5	426.95	93^1_0	b_{2u}
431.6	1765	1745	5.7	(1746)	428.1		$23^1_0 97^1_0$	b_{2u}
429.4	1883	1866	5.7	(1867)	425.6		$23^1_0 115^1_0$	b_{3u}
428.5	1932	1934	29.1	(1935)	424.7		$23^1_0 93^1_0$	b_{2u}
422.7	2252	2251	4.1	(2251)	491.1		$23^2_0 93^1_0$	b_{2u}

[a]Peak positions in the absorption spectrum of $C_{32}H_{14}$ in solid Ne reported by Ehrenfreund et al.[5] [b]Peak position in the fluorescence excitation spectrum of jet-cooled $C_{32}H_{14}$ reported by Amirav et al.[3] [c]Peak positions in wavenumbers relative to the tentatively assigned 0^0_0 band of the $S_3(B_{1g})\leftarrow S_0(A_g)$ transition at 21405 cm^{-1}.[d]Peak positions from the convoluted simulated stick spectrum calculated at the B3LYP-GD3BJ/6-311++G(2d,2p) level. Vibrational wavenumbers were scaled by 0.98. Bands in parentheses were not definitively identified in experiments. [e]Intensities relative to the most intense band at 1619 cm^{-1} (ν_{93}, b_{2u}) in %; only those greater than 1.5% are listed. [f]Harmonic vibrational wavenumbers calculated at the B3LYP-GD3BJ/6-311++G(2d,2p) level and scaled by 0.98. Values for combination bands given in parentheses are the sum of the fundamentals.

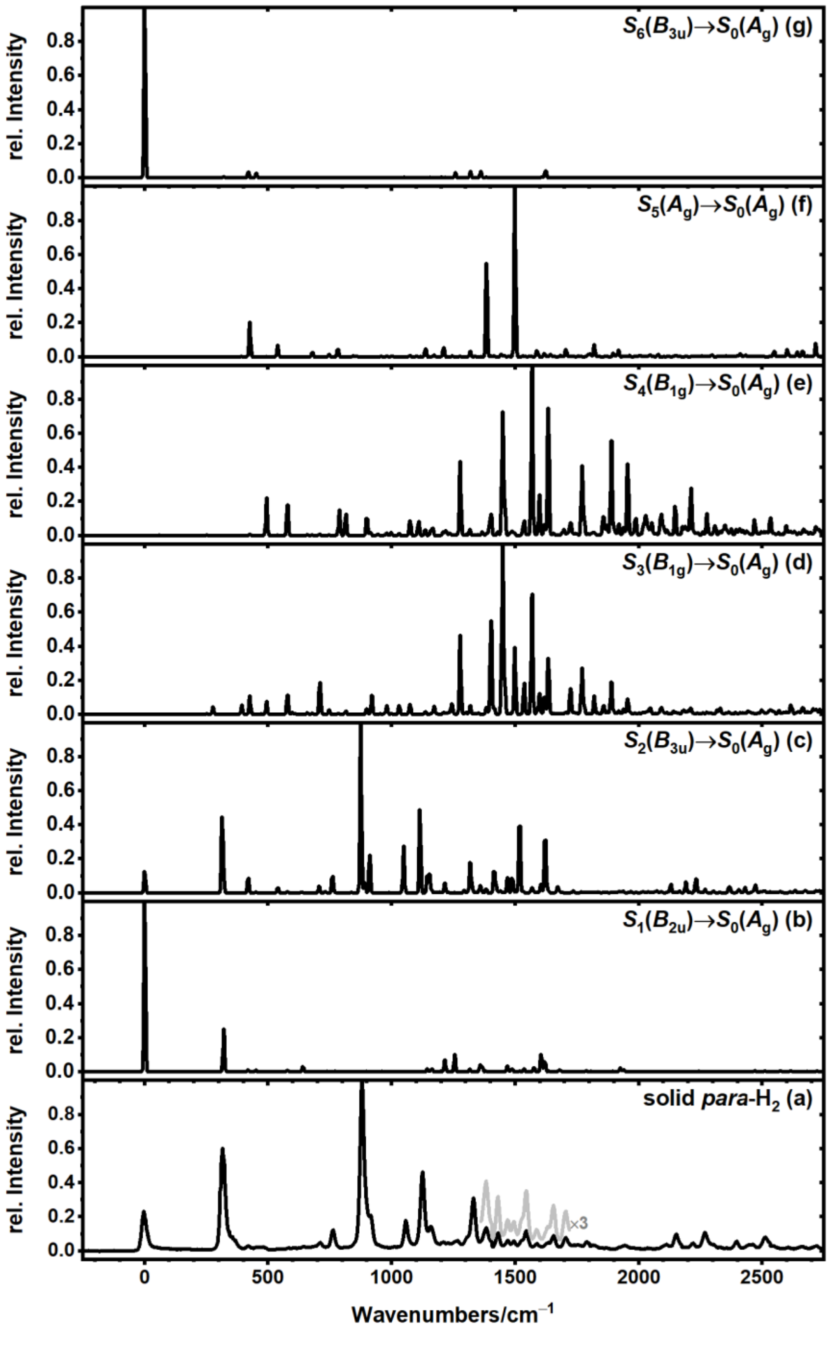

Figure S1. Comparison of experimental dispersed fluorescence spectrum with vibrationally resolved $S_n \leftarrow S_0(A_g)$ (n = 1−6) electronic absorption spectra. The experimental spectrum in (a) is taken from Figure 2a. The spectra were simulated with Franck-Condon Herzberg-Teller calculations according to the optimized geometries and scaled harmonic vibrational wavenumbers of the ground and excited states computed at the (TD-)B3LYP-GD3BJ/6-311++G(2d,2p) level. For a better comparison of the band patterns, the individual spectra have been normalized to their respective most intense bands and aligned with respect to their 0_0^0 band positions. For transition energies, see Table S1. Harmonic wavenumbers have been scaled by 0.980.

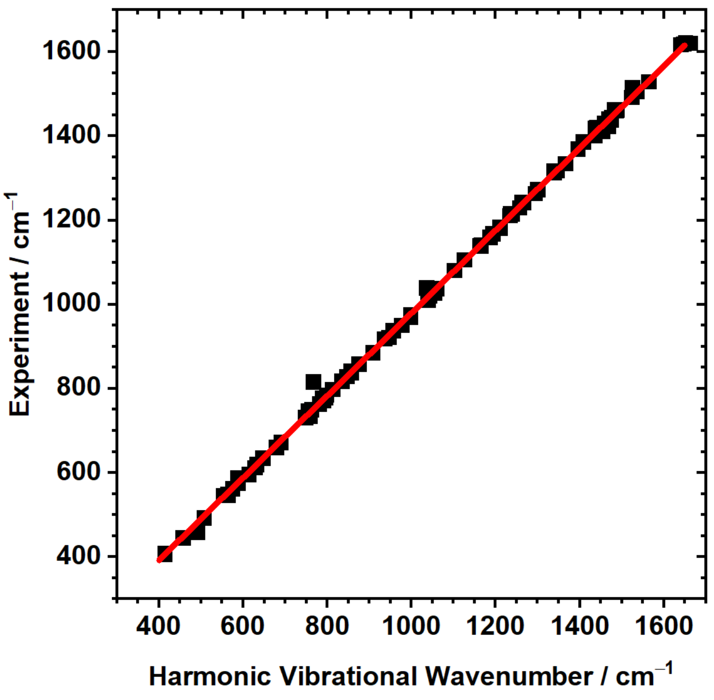

Figure S2. Linear least-squares fit of experimental vibrational wavenumbers vs. calculated harmonic vibrational wavenumbers. The experimental wavenumbers inferred from the IR spectra of stable PAH (fluoranthene, coronene, ovalene, corannulene, sumanene) isolated in solid *para*-H_2 as a function of harmonic vibrational wavenumbers calculated with the B3LYP-GD3BJ/6-311++G(2d,2p) method are plotted. The fitted equation (solid red line) is: $\nu_{scaled} = \nu_{harmonic} \times (0.980 \pm 0.001)$; the listed error represents one standard deviation in fitting.

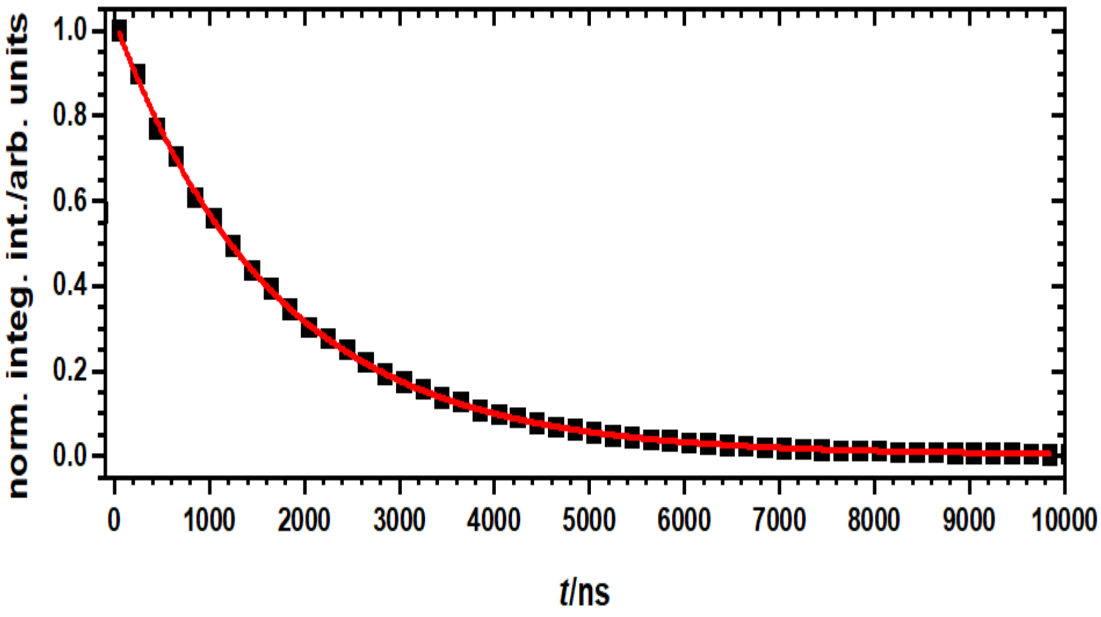

Figure S3. Time-resolved integrated fluorescence emission (black dots) of $C_{32}H_{14}$ isolated in solid *para*-H_2 upon excitation at 22119 cm^{-1} (452.1 nm). Emission, probed in the range 20095–20243 cm^{-1} corresponding to emission from $S_2(B_{3u})$, was detected for 100 ns at 200-ns intervals. An exponential fit (red line) to the equation $y = y_0 + A \exp(-t/\tau)$ gives an emission lifetime $\tau = 1700 \pm 10$ ns; the error limit represents one standard deviation in fitting.

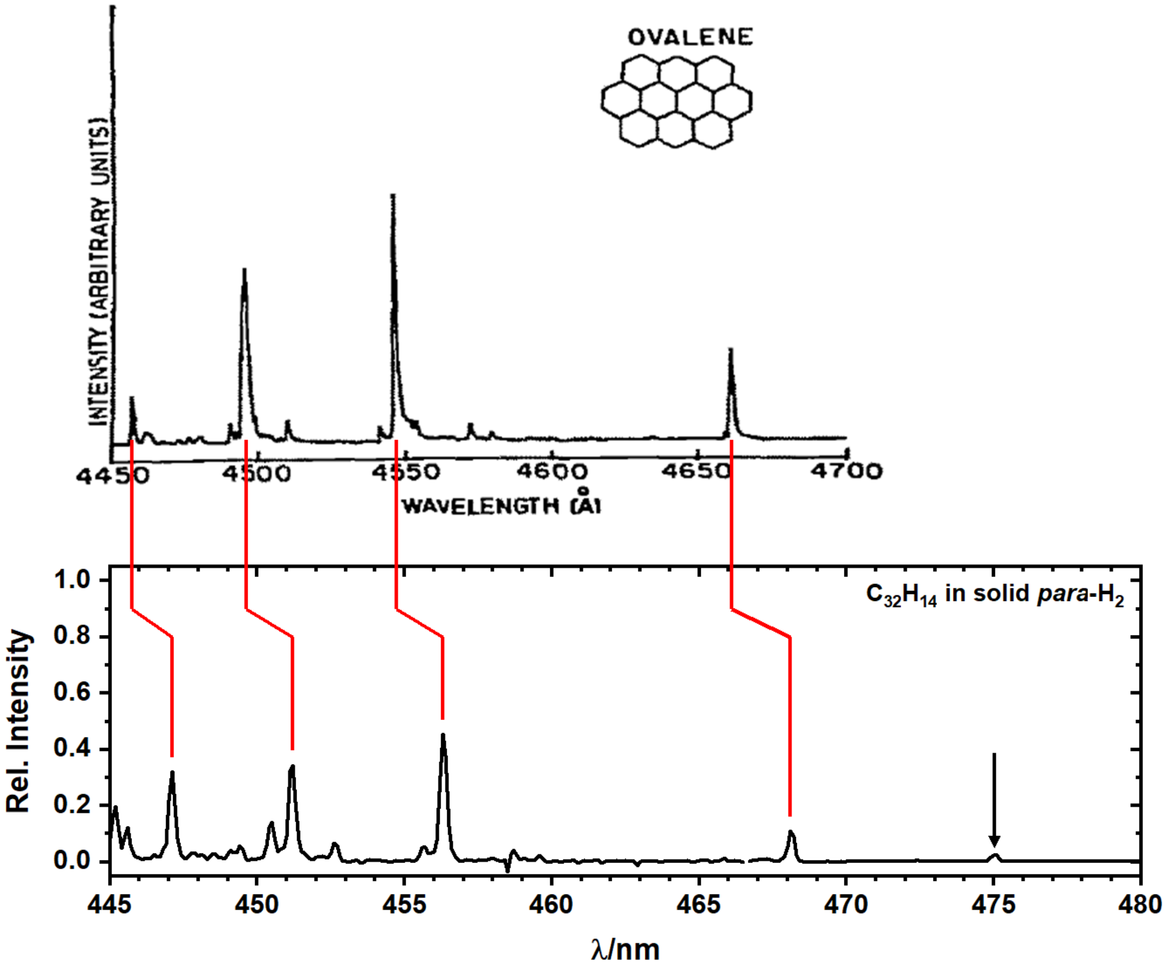

Figure S4. Comparison of observed $S_2(B_{3u})$←$S_0(A_g)$ fluorescence excitation spectrum of $C_{32}H_{14}$ isolated in solid *para*-H_2 with the spectrum of jet-cooled $C_{32}H_{14}$ reported by Amirav et al.[3,4] The red lines indicate the correlation of each band. The reassigned 0_0^0 transition is marked with an arrow. (The top figure is from Amirav, A.; Even, U.; Jortner, J., Intermediate level structure in the $S_2(B_{3u})$ state of the isolated ultracold ovalene molecule, Chem. Phys. Lett. **1980**, 69, 14−17. Copyright, with permission from Elsevier)

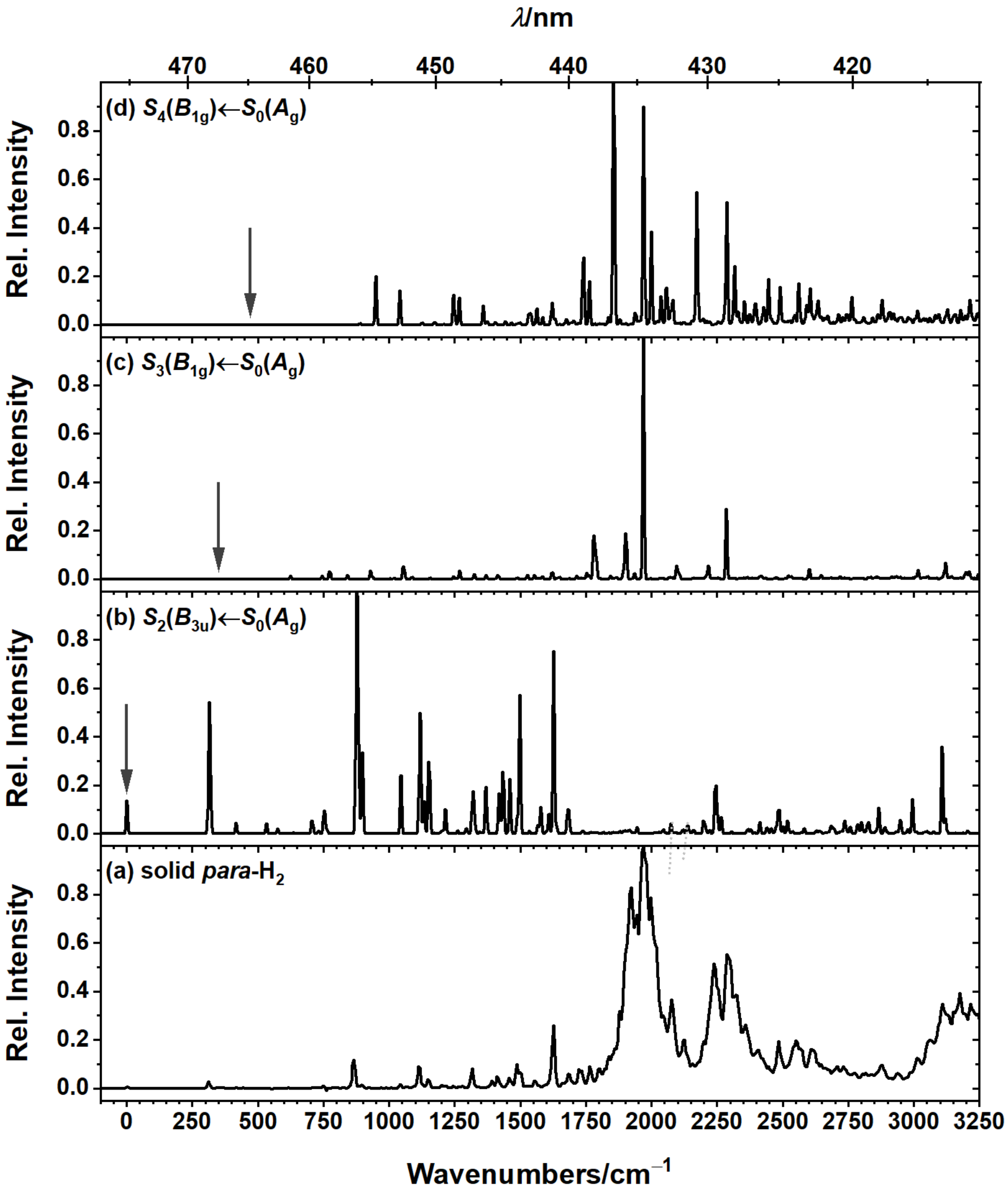

Figure S5. Comparison of experimental fluorescence excitation spectrum with simulated spectra of $S_2(B_{3u})\leftarrow S_0(A_g)$, $S_3(B_{1g})\leftarrow S_0(A_g)$, and $S_4(B_{1g})\leftarrow S_0(A_g)$. (a) Extended fluorescence excitation spectrum of $C_{32}H_{14}$ isolated in solid *para*-H_2; (b) Simulated $S_2(B_{3u})\leftarrow S_0(A_g)$ absorption spectrum; (c) Simulated $S_3(B_{1g})\leftarrow S_0(A_g)$ absorption spectrum shifted by +351 cm^{-1} to align the most intense feature with the most intense band in the experimental spectrum; (d) Simulated $S_4(B_{1g})\leftarrow S_0(A_g)$ absorption spectrum shifted by +472 cm^{-1} to align the intense doublet with the most intense band in the experimental spectrum. The transition origins are indicated with arrows.

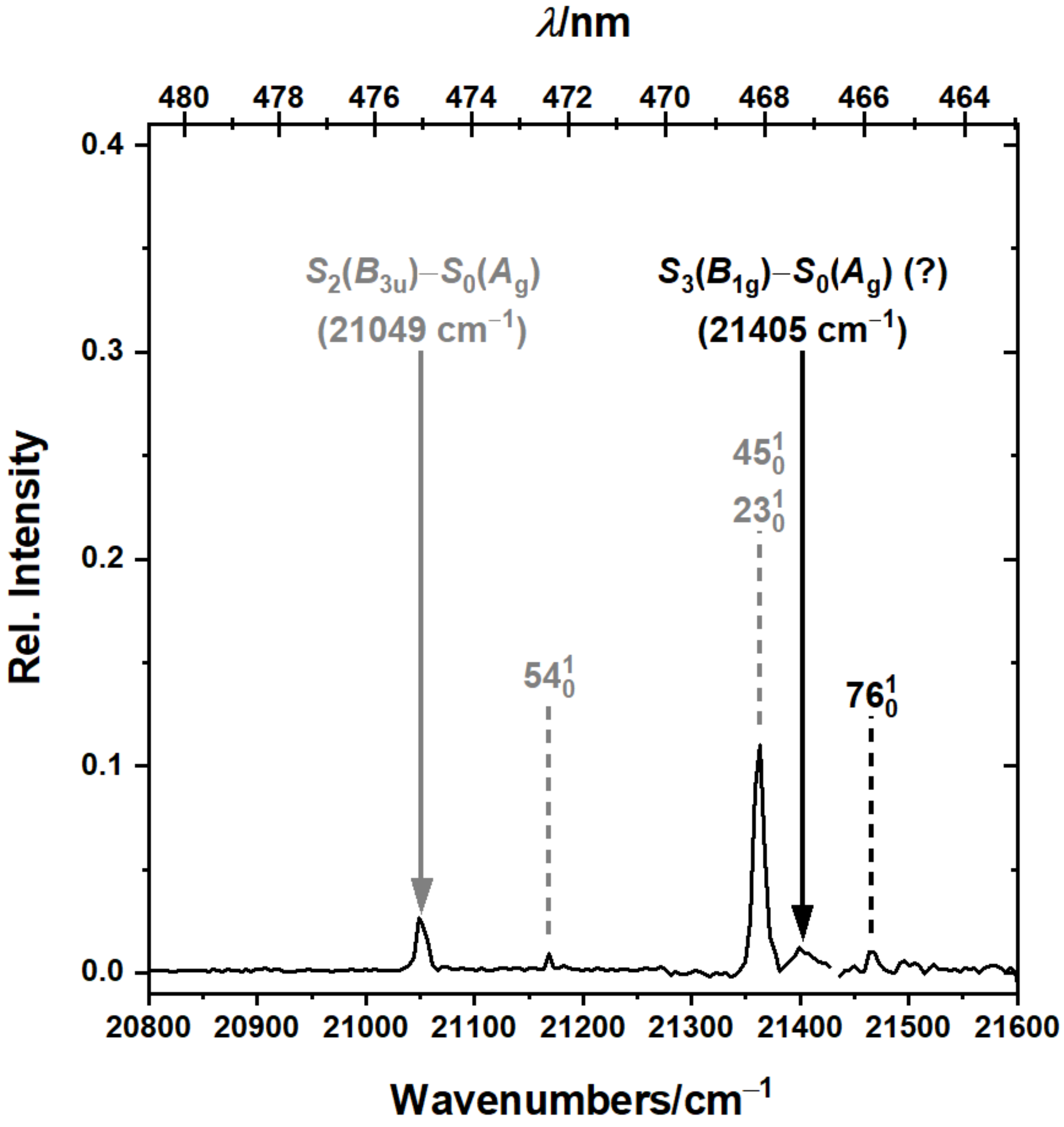

Figure S6. Partial fluorescence excitation spectrum of $C_{32}H_{14}$ isolated in solid *para*-H_2 indicating the 0^0_0 bands of the $S_2(B_{3u})$←$S_0(A_g)$ and $S_3(B_{1g})$←$S_0(A_g)$ transitions. The 0^0_0 band of the $S_2(B_{3u})$←$S_0(A_g)$ transition is at 21049 cm^{-1}. The weak feature at 21405 cm^{-1} is tentatively assigned to the 0^0_0 band of the $S_3(B_{1g})$←$S_0(A_g)$ transition. The intense band at 21363 cm^{-1} has been ascribed to the combination of 45^1_0 and 23^1_0 in the S_2←S_0 absorption spectrum. The sharp but very weak feature at ~21168 cm^{-1}, ~119 cm^{-1} from the 0^0_0 band can be tentatively assigned to the S_2←S_0 54^1_0 transition (b_{2g}, 117 cm^{-1}) , predicted by our calculations with a relative intensity of 0.08 %. The very weak peak at ~21466 cm^{-1}, ~61 cm^{-1} from the 0^0_0 band of the S_3 ←S_0 transition, is consistent with an extremely weak feature (relative intensity 0.01 %) in the predicted S_3←S_0 absorption at 62 cm^{-1} corresponding to 76^1_0 (a_u). Features associated with the S_2←S_0 transition are labelled in grey, those associated with the S_3←S_0 transition in black.

References

1. Perera, M.; Tom, B. A.; Miyamoto, Y.; Porambo, M. W.; Moore, L. E.; Evans, W. R.; Momose, T.; McCall, B. J. Refractive Index Measurements of Solid Parahydrogen. *Opt. Lett.* **2011,** *36*, 840-842.
2. Berlman, I. B., *Handbook of Fluorescence Spectra of Aromatic Molecules*. 2nd ed.; Academic Press: New York, 1971.
3. Amirav, A.; Even, U.; Jortner, J. Excited-State Dynamics of the Isolated Ultracold Ovalene Molecule. *J. Chem. Phys.* **1981,** *74*, 3745-3756.
4. Amirav, A.; Even, U.; Jortner, J. Intermediate Level Structure in the S2 State of the Isolated Ultracold Ovalene Molecule. *Chem. Phys. Lett.* **1980,** *69*, 14-17.
5. Ehrenfreund, P.; D'Hendecourt, L.; Verstraete, L.; Leger, A.; Schmidt, W.; Defourneau, D. Search for the 4430 A DIB in the Spectra of Coronene Cation and Neutral Ovalene. *Astron. Astrophys.* **1992,** *259*, 257-264.
6. Ruiterkamp, R.; Halasinski, T.; Salama, F.; Foing, B. H.; Allamandola, L. J.; Schmidt, W.; Ehrenfreund, P. Spectroscopy of Large PAHs. *Astron. Astrophys.* **2002,** *390*, 1153-1170.
7. Kropp, J. L.; Stanley, C. C. The Temperature Dependence of Ovalene Fluorescence. *Chem. Phys. Lett.* **1971,** *9*, 534-538.
8. Liang, J.; Feng, X.; Hait, D.; Head-Gordon, M. Revisiting the Performance of Time-Dependent Density Functional Theory for Electronic Excitations: Assessment of 43 Popular and Recently Developed Functionals from Rungs One to Four. *J. Chem. Theory Comput.* **2022,** *18*, 3460-3473.
