# Supplementary material for: Electronic Spectroscopy of Ovalene: Reassignment of the S2(B3u)– S0(Ag) Transition
Source: J Phys Chem Lett. 2024 Oct 16;15(42):10696–702. doi: 10.1021/acs.jpclett.4c02494 (PMC11514017; doi:10.1021/acs.jpclett.4c02494)
Supplement: Supplementary file 2 — jz4c02494_si_002.pdf [file jz4c02494_si_002.pdf]

jz-2024-02494b.R1

Name: Peer Review Information for "Electronic Spectroscopy of Ovalene: Reassignment of the <i>S</i>₂(<i>B</i>_{3u})−<i>S</i>₀(<i>A</i>_g) Transition"

First Round of Reviewer Comments

Reviewer: 1

Comments to the Author

This manuscript reports the fluorescence spectra of ovalene isolated in solid parahydrogen. Based on spectral analysis, the authors conclude that the S1-S0 spectra previously reported in a supersonic jet should be reassigned to the S2-S0 transition, and they have reassigned the origin band wavenumber accordingly. Additionally, they estimate the S2-S3 energy gap from the spectral features for the first time. Solid parahydrogen has primarily been used for infrared spectroscopy, and this group is the first to extend its application to electronic spectroscopy. They have applied this technique to planar polycyclic aromatic hydrocarbons, which are considered potential carriers of the diffuse interstellar band. The reported spectra are clear, and the assignments, which are based on quantum chemical calculations, are reasonable. The agreement between the observed and simulated spectra is remarkably good for both dispersed fluorescence and excitation spectra. The manuscript also adequately references previous works, and the writing is clear and well-organized. I recommend this manuscript for publication after minor revisions.

(1) The fluorescence emission spectra in Figure 2 were obtained using 331.0 nm excitation, but there is no explanation provided regarding this excitation wavelength. Since the excitation spectra in Figure 3 show multiple intense peaks between 475 nm and 440 nm in the S2-S0 band, it would be beneficial to include the fluorescence emission spectra corresponding to the S2-S0 excitation peaks.

Reviewer: 2

Comments to the Author

This paper continues the excellent series of spectroscopic contributions from the Lee group, where large PAH molecules have been placed in hydrogen crystals. The present contribution purports to

reassign the S1 and S2 states of ovalene. However, this is problematic. It is evident that internal conversion and non-radiative decay occur within the crystal, because the emission spectrum has been generated using UV excitation, and emission is solely from the v=0 level of the S1 state. Here it is assigned to S2. If it were S2, then why wouldn't there be decay to S1? I have no doubt that what was calculated S2 is the correct state, but offer a very simple explanation:

In naphthalene, TDDFT gets the S1 and S2 states calculated around the wrong way! I am sure this is happening here. The authors have simply calculated S1 as S2. A calculation which allows for double excitations would restore the correct state ordering. I know this is difficult for ovalene, but if the authors confirm that their method also gets naphthalene wrong, then they will be satisfied.

The only other comment is to pay attention to the spelling of Herzberg!

Author's Response to Peer Review Comments:

We have revised the manuscript according to the reviewers' comments. The detailed description of the revision is attached.

We appreciate very much the valuable comments and suggestions from the reviewers.

Below are the detailed responses to the reviewer's comments on the manuscript. The reviewer comments are in black, our responses are listed in blue font after each comment, and the revised text are highlighted in blue.

Reviewer: 1

This manuscript reports the fluorescence spectra of ovalene isolated in solid parahydrogen. Based on spectral analysis, the authors conclude that the S1-S0 spectra previously reported in a supersonic jet should be reassigned to the S2-S0 transition, and they have reassigned the origin band wavenumber accordingly. Additionally, they estimate the S2-S3 energy gap from the spectral features for the first time. Solid parahydrogen has primarily been used for infrared spectroscopy, and this group is the first to extend its application to electronic spectroscopy. They have applied this technique to planar polycyclic aromatic hydrocarbons, which are considered potential carriers of the diffuse interstellar band. The reported spectra are clear, and the assignments, which are based on quantum chemical calculations, are reasonable. The agreement between the observed and simulated spectra is remarkably good for both dispersed fluorescence and excitation spectra. The manuscript also adequately references previous works, and the writing is clear and well-organized. I recommend this manuscript for publication after minor revisions.

(1) The fluorescence emission spectra in Figure 2 were obtained using 331.0 nm excitation, but there is no explanation provided regarding this excitation wavelength. Since the excitation spectra in Figure 3 show multiple intense peaks between 475 nm and 440 nm in the S2-S0 band, it would be beneficial to include the fluorescence emission spectra corresponding to the S2-S0 excitation peaks.

Response/Revisions: We agree with the reviewer that displaying the dispersed fluorescence spectrum recorded upon excitation at 331.0 nm, i.e. to the S_6 state of ovalene, has been a somewhat odd choice motivated solely by the chronology of the experiments. During our work, we recorded dispersed fluorescence spectra upon excitation at various wavelengths corresponding to the S_2 to S_6 states; these spectra closely resemble each other with respect to relative peak intensities and peak positions, as expected for matrix-isolated species of which vibrational relaxation and internal conversion are usually fast. To avoid unnecessary confusion, we have now replaced the dispersed fluorescence spectra depicted in Figure 2 and Figure S1 with a dispersed fluorescence spectrum of ovalene isolated in solid *para*-H_2 recorded upon excitation at 431.5 nm and modified the Figure captions and the main test on page 6 accordingly. It now reads "Upon excitation of this matrix at 22660 cm^{-1} (431.1 nm), we observed […]"..

Reviewer: 2

This paper continues the excellent series of spectroscopic contributions from the Lee group, where large PAH molecules have been placed in hydrogen crystals. The present contribution purports to reassign the S1 and S2 states of ovalene. However, this is problematic. It is evident that internal conversion and non-radiative decay occur within the crystal, because the emission spectrum has been generated using UV excitation, and emission is solely from the v=0 level of the S1 state. Here it is assigned to S2. If it were S2, then why wouldn't there be decay to S1? I have no doubt that what was calculated S2 is the correct state, but offer a very simple explanation:

In naphthalene, TDDFT gets the S1 and S2 states calculated around the wrong way! I am sure this is happening here. The authors have simply calculated S1 as S2. A calculation which allows for double excitations would restore the correct state ordering. I know this is difficult for ovalene, but if the authors confirm that their method also gets naphthalene wrong, then they will be satisfied.

Response/Revisions: Yes, TD-DFT indeed gets the wrong order for the S1 and S2 states of naphthalene; this is also true for the B3LYP-GD3BJ/6-311++G(2d,2p) method employed in this work. Nonetheless, this is not necessarily true for all molecules of D_{2h} symmetry. In 2019, Benkyi et al. (*PCCP* **2019**, 21, 21094) computed the lowest two excited states (transition energies and oscillator strength) for different acenes – naphthalene, anthracene, and pentacene – and pyrene by TD-DFT and CC2. From a comparison of absorption spectra simulated on the basis of these calculations to experimental data, they concluded that CC2 is capable of capturing correctly the order of excited states for the selected molecules; TD-DFT, on the contrary, predicts an inverse order of S1 and S2 states for naphthalene and pyrene, but is consistent with CC2 for the larger acenes, anthracene and pentacene.
Whether TD-DFT is capable of capturing the correct order of the S1 and S2 states of ovalene, ultimately can only be confirmed by higher level calculations – this, however, is beyond the scope of the present work – or by successful experimental observation of emission at lower energies consistent with a lower lying S1 state.

We are now addressing these concerns in an additional paragraph on page 8, stating "To observe emission from the S_2 state rather than the S_1 state for a molecule isolated in a matrix is a little unusual, if one considers the rapid internal conversion typically observed in matrices. It is known that TD-DFT calculations predict an inverse order for the S_1 and S_2 states of naphthalene and pyrene, both belonging to point group D_{2h}; this is also the case for the TD-B3LYP-GD3BJ/6-311++G(2d,2p) method employed in this work. Comparing the results of TD-DFT and CC2 calculations for the S_1 and S_2 states of pyrene and three acenes: naphthalene, anthracene, and pentacene, Benkyi et al.[31] found an inversed order of excited states S_1 and S_2 in the TD-DFT calculations for only naphthalene and pyrene; for the larger acenes, TD-DFT results were consistent with the results from CC2 calculations, which agreed well with previously reported experimental data. Whether our TD-DFT calculations predict the correct

order of the S_1 and S_2 states of ovalene cannot be definitely concluded on the basis of the results presented here alone; either higher-level calculations or the experimental confirmation of the existence of a lower-lying electronic excited (S_1) state would be required to confirm the order of these two states. Nevertheless, our results indicate that the excited state should be a B_{3u} state rather than a B_{2u} state." and added the publication of Benkyi et al. as reference 31.

To avoid confusion due to the order of the electronic excited states, we now added the symmetry of each electronic state when needed.

The only other comment is to pay attention to the spelling of Herzberg!

We corrected the spelling at all occurrences on page 6 (Figure caption and main text), page 10 (Figure caption), and page 13.
